# Sulphite of Soda in Acute Dysentery

**Published:** 1876-04

**Authors:** 


Sulphite of Soda in Acute Dysentery.—Dr. John B. C. Gazzo, of Thebodeau, La Fourche Parish, La., wishes to call the attention of the profession to the virtues of sulphite of soda as a remedy for dysentery. He employs it in the following manner:

℞—Soda Sulphis, . . . . . . ℥ i.

Acid Salicylic, . . . . . . . ʒ ij.

Aquæ Destil. . . . . . . . ℥ v.

Syrup. Rosa Galli., U. S. P.,

Sinc. Opii fl. aa. . . . . . . . ℥ ss.

Syrupi Vanilla, . . . . . . . ℥ i.

Misce. Fiat mistura.

The mixture was given in half tablespoonful doses, with an equal quantity of fresh milk every half hour. On account of its antiseptic and disinfectant properties he believes it can be employed in the form of an enema, and that it will not only destroy or annihilate the offensive, decayed, and putrid evacuations, but will also give considerable relief to the patient. The ordinary means for keeping up the circulation are also employed, such as stimulating antispasmodic liniments to the bowels and limbs. All of his cases did well.—*Medical Record.*

How to Remove Stains from Steel Knives.—The very best way to clean a stained steel knife, is to cut a solid potato in two, dip one of the pieces in brick-dust, such as is usually used in knife-cleaning, and rub the blade with it.—*Med. Brief.*

